# Characterization of the Stretch Flangeabitity of High-Strength Bainitic Steel: The Significance of Variant Pairs

**DOI:** 10.3390/ma15010276

**Published:** 2021-12-30

**Authors:** Zhiquan Wang, Zifeng Guo, Chengjia Shang, Bin Chen, Yajun Hui

**Affiliations:** 1 Collaborative Innovation Center of Steel Technology, University of Science and Technology Beijing, Beijing 100083, China; eleprexquan@163.com; 2 Sheet Metal Research Institute, Shougang Group Co., Ltd. Shougang Research Institute of Technology, Beijing 100043, China; guozifeng5588@163.com (Z.G.); ustbchenbin@163.com (B.C.); huiyajun2013@sina.com (Y.H.); 3 Shougang Qian’an Iron &Steel Co. Ltd., Qian’an 064404, China

**Keywords:** hole expansion ratio, variant pairs, CP group, Bain group, texture, dislocation

## Abstract

Variant pairs have an indispensable function on mechanical properties such as low impact toughness. Therefore, it was assumed that they would also affect the HER (Hole Expansion Ratio, an indicator to evaluate stretch flanging performance). To clarify this, a comprehensive analysis of the common influential factors in an 800 MPa grade low carbon micro-alloyed steel, i.e., the retained austenite, the M/A (Martensite/Austenite) island, the titanium precipitations, the grain diameter, the density of high angle grain boundaries and the textures, was first conducted. It was found that they did not match well with the HER, suggesting that they were not the governing factor for HER in this steel. However, the dominating crystallography groups and the variant pairing results indicated that they fitted well with the HER. In the samples with high HERs, the CP (Close Packed) groups dominated the transformation, wherein one individual CP group consisted of two or more Bain groups, whereas it evolved into the domination of joint CP groups and Bain groups for the low HER sample. Further analysis on the variant pairing features indicated that a correlation occurred between the HER and the high angle variant pairs. In the steels with high HERs, high-angle variant pairs of V1/V2, V1/V3 that transformed from the same CP group, particularly of V1/V2 pair, were mostly generated. They turned to V1/V9, V1/V10, V1/V12, V1/V15, V1/V17, and V1/V18 pairs from differential CP groups, especially the V1/V12 and V1/V15 pair for low-HER steel. This result showed that V1/V2, V1/V12, and V1/V15 might have accounted most for the HER in this steel. The underlying reason was that the V1/V2 pair was specialized in supplying a slip passage for dislocation transmission across a grain boundary with little resistance, whereas the dislocation transmission ability for V1/V12 and V1/V15 pair was particularly poor. Thus, to efficiently enhance the HER, one should regulate the variant pairs by augmenting the V1/V2 fraction and suppressing the formation of the V1/V12 and V1/V15 pair.

## 1. Introduction

The hole-expansion ratio (HER) is a measure of stretch flangeability performance for automotive structural steels [1]. With the advanced-high strength steels nowadays becoming extensively employed in fabricating automobile structural parts to improve fuel efficiency and to eliminate gas emissions [2,3,4,5,6,7,8], the cracking caused by dislocation pile-ups (which introduces stress concentration during deformation) or strength difference between differential microstructures during stretch flangeability operation of AHSS has been the major restriction in their use [2,3,4]. Enhancing the HER and figuring out the controlling factors to the HER are of great significance. According to previous studies [2,3,4,5,6,7,8,9,10,11,12,13,14,15,16,17,18,19,20,21,22,23], the influential aspects of HER include the deformation details, the punch geometries, the tensile properties, and the microstructure of the steel sheet [4,5,6,7,8,9,10,11,12,13,14,15,16,17,18,19,20,21,22,23]. However, most steel researchers focus on the material itself, and a considerable number of experimental and simulation works [4,9,11] have been done on the effect of the microstructure to the HER, respectively, for the aspects of the phases constituting the microstructure [5,10,12,14] the anisotropy [15,16,17,18,19,20,21], the austenite stability [22], strain hardening exponent (n) [17], stress state [23], etc. Undoubtedly, all these studies will assist developers in developing a high HER steel to some extent. However, no general conclusions on the controlling factor to HER have been obtained. It is still unlikely to precisely design the target HER or predict the HER when considering the above factors only, suggesting that the factors affecting the HER should not be all-inclusive as in the previous studies. Considering that the microstructure of the steels is intrinsically crystal-like, the crystallographic features should exert a non-ignorable action on the HER. Although present studies [15,16,17,18,19,20,21] have concluded that the crystallographic characteristics (primarily for anisotropy or textures) will alter the stretch-flange-formability, the intrinsic aspect of variant pairing characteristics was omitted in these researches as a result of the absence of deep analytical methods. However, it is possible [24,25,26,27] now.

According to K-S (${\left(111\right)}_{\gamma }//{\left(011\right)}_{\alpha }$,$\;\bar{1}01_{\gamma }//\bar{1}\bar{1}1_{\alpha }$) or near K-S orientation relationship, a total of twenty-four variants and sixteen groups of variant pairs in steels are likely to be transformed. Constrained by the OR (orientation relationship) to the parent grain, the orientation relationship of a given variant pair is fixed regardless of the orientation of the prior austenite grain. This fixed orientation relationship of a variant pair would generate some collaborative effects on the mechanical properties [24,25,26,27], and also on the stretch flangeability. It can also be speculated that the influence of each individual variant pair on the stretch flanging performance would be dissimilar as the orientation relationship for each pair varies. Once the impacts caused by each individual variant pair are clarified, regulating stretch-flange formability will be more achievable.

Hence, the authors in this article first discuss the factors influencing the HER by employing an 800 MPa grade low carbon low alloyed steel in the traditional way, and then, determine the relationship of the dominant crystallography groups and variant pair fractions with the HER.

## 2. Experimental Procedure

The steel plates in this study were 800 MPa grade low carbon (0.05–0.08 w.t% (C)) alloyed steel fabricated for commercial applications (Shougang Qian’an Iron &Steel Co. Ltd., Qian’an 064404, Hebei, China). The steel was alloyed by (Cr) + (Ti) (Shougang Qian’an Iron &Steel Co. Ltd., Qian’an 064404, Hebei, China) with a total amount of 0.2–0.5 w.t% in composition, and the (Mn) (Shougang Qian’an Iron &Steel Co. Ltd., Qian’an 064404, Hebei, China) was 1.2–1.8 w.t%. The detailed chemical compositions are listed in Table 1. Three steel plates with a thickness of 3.2 mm were fabricated in the plant. They were hot-rolled with the same rolling parameters except for the middle temperature (Mt) during strip laminar cooling, which was 520 °C (A), 540 °C (B), and 560 °C (C). To accurately calculate the OR, non-deformed austenite was also formed by direct cooling without rolling after the same heat treatment process, assuming that the OR does not change as a result of prior deformation as Miyamoto reported [28,29]. After cooling, the samples for hole expansion tests were cut from the cooled steel plates to be 3.2 × 92 × 92 mm^3^ in size with a punched hole 10 mm in diameter in the center. Hole expansion tests were conducted according to a Chinese GB/T 15825.4-2008 standard on the ZWICK BUP400 forming test machine (Zwick, Ruhr-gebiet, Germain). The driving speed of the conical punch was 0.3 mm/s. The punch would continue until the crack on the hole edge penetrated through the sample, which could be observed by the optical instrument. For each valid tested sample, the average diameter of the original hole *D*_0_ and ruptured hole *D_r_* was both determined by measurement in three directions that cross 120° to each other. Using the average diameter, the hole expansion ratio λ could be calculated using the following equation:

(1) $$\lambda =\frac{\bar{D_{r}}-\bar{D_{0}}}{\bar{D_{0}}}\times 100\%$$

Once the HER was calculated, the samples for tensile testing for these steel plates were cut at the position of 1/4 to the plate width in the longitudinal (L) direction. They were measured at room temperature (25 °C) using standard tensile samples machined to 3.2 mm in thickness and 80 mm gauge length. After punching, three specimens for microstructure analysis were cut separately from the edge of the punched samples in the longitudinal direction. Optical microscopy (OM) (Olympus Corporation, Tohoku, Japan) and electron back-scattering diffraction (EBSD) (Oxford Instruments, Oxford, England) were used to study the microstructure evolution in terms of morphology and crystallography. The volume fraction of the retained austenite was determined by using a D_MAX_-RB 12 kV X-ray diffractometer (Bruker AXS, Massachusetts, America) with Cu Kα radiation under the following conditions: acceleration voltage, 40 kV; current, 150 mA; step, 0.02°. The X-ray diffraction data were analyzed by Jade 6.5. It is quantified using the formula suggested in reference [30] as follows:

(2) $$V\gamma =\frac{1.4I\gamma }{I\alpha +1.4\;I\gamma }$$

where Vγ is the volume fraction of retained austenite; Iγ is the average integrated intensity of the austenite at {200}γ, {220}γ, and {311}γ plane diffraction peaks; and Iα is the average integrated intensity of ferrite at {200}α and {211}α plane diffraction peaks. 

The specimens were mechanically polished using standard metallographic procedures and etched with 4% nital (Olympus Corporation, Tohoku, Japan) for OM observation. The extraction replicas were prepared by evaporating an amorphous carbon film onto the bulk sample that had been polished and lightly pre-etched. The specimens were observed in TEM (FEI F20) (FEI, Oregon, America) operated at 200 kV. The M/A continents were characterized by LePera’s reagents (Olympus Corporation, Tohoku, Japan). For EBSD and XRD tests, the specimens were re-prepared with a final mechanical and chemical polishing using 0.05 μm colloidal silica (Oxford Instruments, Oxford, England). EBSD data were obtained by using TESCAN MIRA3 LMH Scanning Electron Microscope (Tescan, Brno, Czech) equipped with an Oxford EBSD detector (Oxford Instruments, Oxford, England)under the following conditions: acceleration voltage, 20 kV; working distance, 16 mm; tilt angle, 70°; step size, 0.08 μm. Channel 5 software (version 5.12) from Oxford-HKL, Oxford Instruments, Oxford, England, as employed for post-processing orientation data. Matlab software, Company name, Location was used for the quantitative calculation of orientation relationship and variant pair fractions. 

## 3. Results and Discussions

### 3.1. The Mechanical Properties and the Hole Expansion Ratio

The hole expansion ratio for the three steel plates is 87.4% (A), 71.3% (B), and 41.8% (C), respectively. The corresponding mechanical properties are listed in Table 2. It seemed that the HER exhibited a strong positive correlation to the total elongation, which agrees with the results in reference [4]. Though the HER was negatively related to the yield strength and tensile strength, their relationship to the HER was not as serious as that to the total elongation.

### 3.2. Morphological Structures

**Figure 1:**
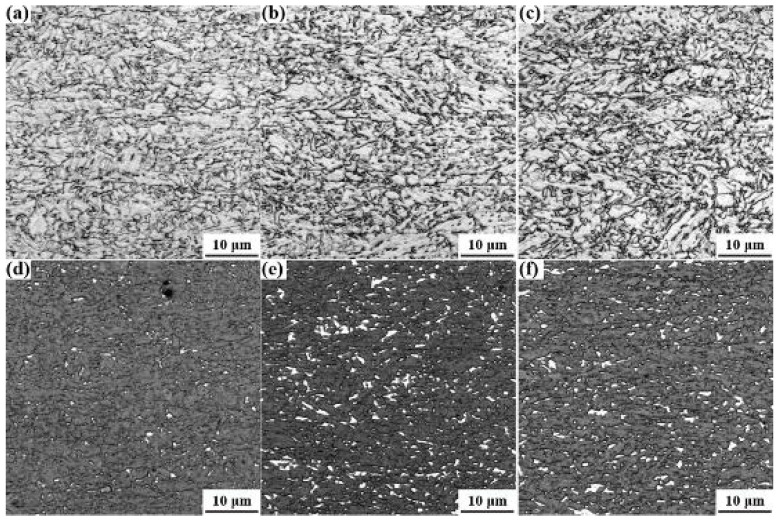
OM micrograph of the microstructure of the best HER (**a**), the worst HER (**b**), and the second-best HER (**c**) and the corresponding M/A continents distribution map (**d**–**f**) to (**a**–**c**), respectively.

**Figure 2:**
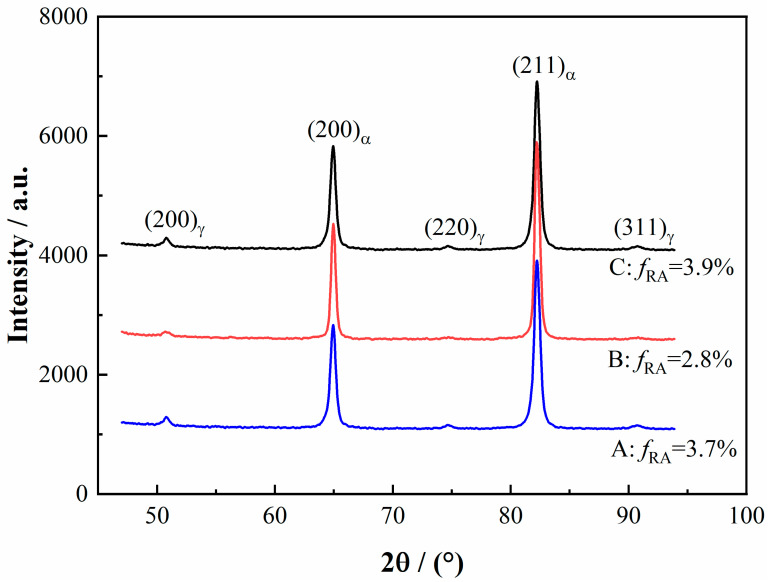
XRD spectra illustrating different volume fractions of retained austenite in specimen A, B, and C.

Optical micrographs in Figure 1 show that the microstructures of these specimens were granular bainites contained M-A continents. The volume fraction of M-A contents in the specimen with the best HER (0.7%) was considerably lower than that in the worst HER specimen (4.6%) and the second-best HER specimen (5.1%). X-ray diffraction results (Figure 2) revealed that the volume fraction of the retained austenite in these specimens was nearly the same, which was around (3.7 ± 0.1)%. It was a bit low in the specimen with the second-best HER, which was (2.8 ± 0.1)%. Due to the level of accuracy of XRD results being about ±3%, the fraction of the retained austenite in all these specimens was only for reference, since the fraction was low. To some extent, we can say that the form of the austenite in the specimen with the best HER was retained austenite, whereas it participated in the formation of the M-A continents in specimens B and C. Sugimoto et al. [31] stated that the retained austenite to martensite transformation during deformation was detrimental to the HER, which was attributed to the fact that the martensite was a hard phase. De Moor et al. [32] concluded that the retained austenite would transform into hard martensite during deformation in their steel as well. Kim et al. [33] also suggested that the retained austenite possessed low stability and would transform to martensite during punching in 0.22C–3.79Mn–1.48Si–0.98Cr quenched and partitioned steel. Since the chemical compositions of the austenite stabilization elements in this steel were lower than in Kim’s research [33] and the studied steel experienced no partition process, the stability of the retained austenite would be alleviated compared to that in Kim’s research. As a result, the retained austenite in this studied steel should be transformed into martensite, and the HER should be degraded. However, the HER of the specimen with the highest volume fraction of retained austenite ((3.7 ± 0.1)%) was the best, implying that there were other issues concerning the HER. Additionally, the M-A continents were hard phase and were also detrimental to the HER. Therefore, it can be predicted that the HER of specimens B and C should be almost equal. However, this was contradictory to the actual results, wherein specimen C with a similar high fraction of M-A continents exhibited exceedingly higher HER (71.3%) than that in specimen B. This implied that the M-A continents should not be the critical issue in controlling the HER in this steel, as with the retained austenite. Though the M-A continents and retained austenite were not the controlling factors in this steel, they might attenuate the stretch flangeability to some extent. 

**Figure 3:**
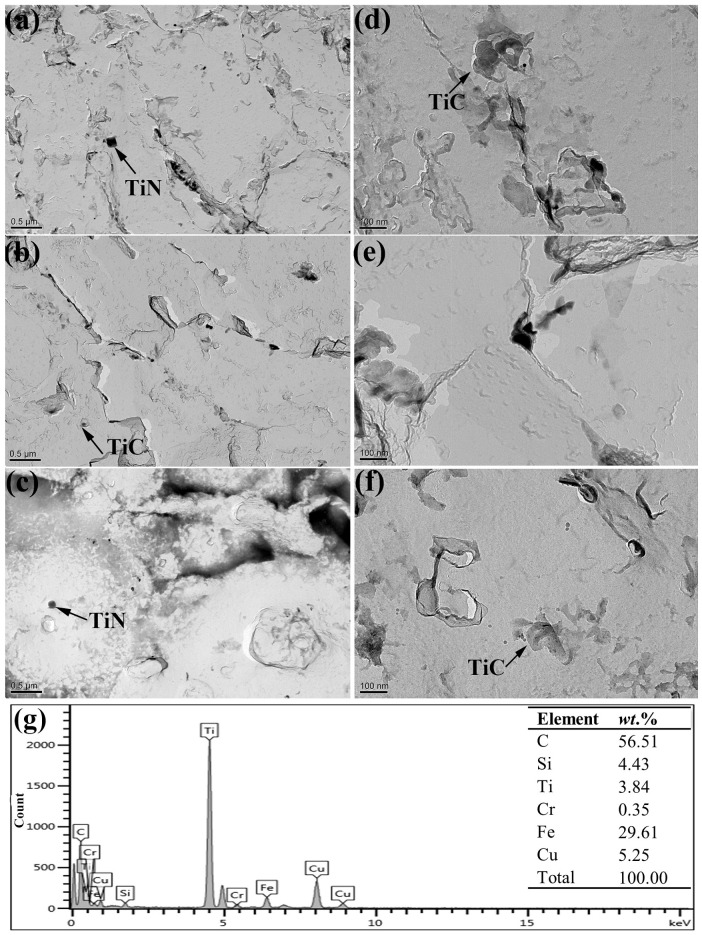
TEM micrographs showing the precipitations of specimen A (**a**,**d**), specimen B (**b**,**e**), specimen C (**c**,**f**), and the corresponding EDS results (**g**).

Figure 3 displays the distribution of the precipitations in these specimens and the EDS results of the precipitate that the black arrow in Figure 3d) indicated, which can be used to infer that the precipitates were TiN (TiN precipitates were often square in shape) and TiC (identified by EDS results). Owing to the low curling temperature of 450 °C, the amounts of TiN and TiC precipitates were all small. Therefore, the effects of precipitations on the HER should be very small. 

### 3.3. EBSD Analysis of Grain Size and Angle Grain Boundary Distribution

**Figure 4:**
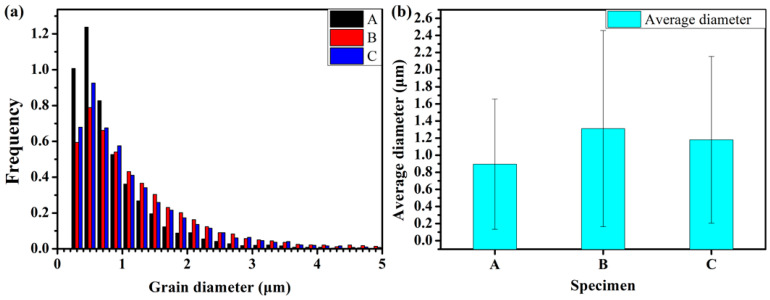
Statistical results of the grain size distribution of these three specimens (**a**) and the average grain diameter (**b**).

Figure 4 illustrates the grain size (defined by the boundaries misoriented over 5°) distribution of these samples, from which we can conclude that specimen A had the smallest grains and the highest HER. It seems that the HER varied negatively with the grain size in this steel. However, specimen B, with a lower grain size, had lower HER than specimen C. These results indicate that the controlling factor of the HER was not the grain size, though it seemed that lowering the grain size should improve the HER. 

**Figure 5:**
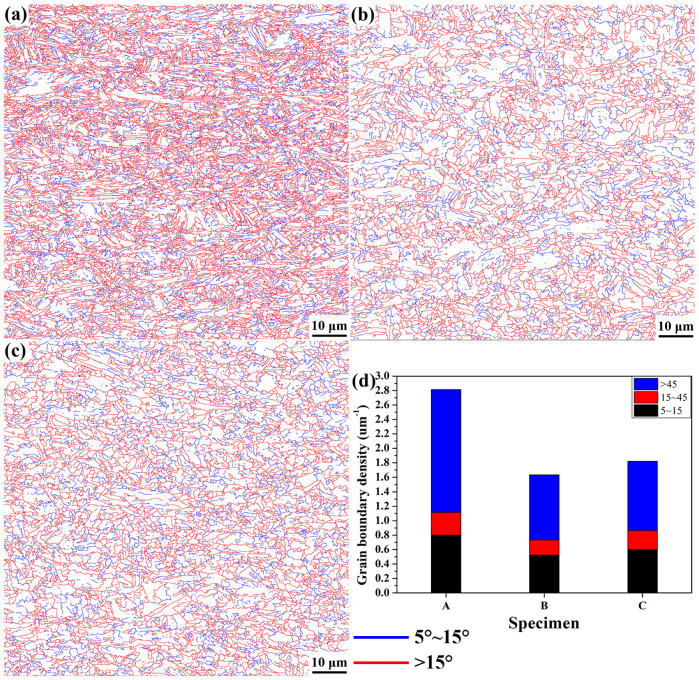
The distribution of grain boundaries (θ > 5°) of specimen A (**a**), specimen B (**b**), specimen C (**c**), and the absolute density of boundaries (**d**).

Figure 5 shows the maps of the distribution of grain boundaries (θ > 5°). It can be clearly seen that the density of grain boundaries in specimen A was exceedingly larger than that in specimens B and C. It was further verified that the grain size of specimen A was the smallest, and specimen B had the largest grain size. The statistical results of the density of boundaries in Figure 5d reported that the densities of low and high angle grain boundaries in specimen A were all greater than in specimens B and C. It seems that the influence of the grain boundaries on HER was analogous to that of the grain size. No apparent correlation existed between the density of grain boundaries and HER as high HER was achieved simultaneously in samples with high (specimen A) and low (specimen B) density of grain boundaries.

### 3.4. Texture Analysis

**Figure 6:**
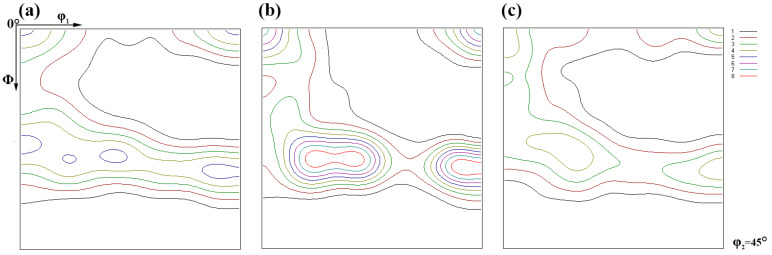
ODF image sectioned at $\varphi_{2}=45{}^{\circ}$ of specimen A (**a**), specimen B, (**b**) and specimen C (**c**).

**Figure 7:**
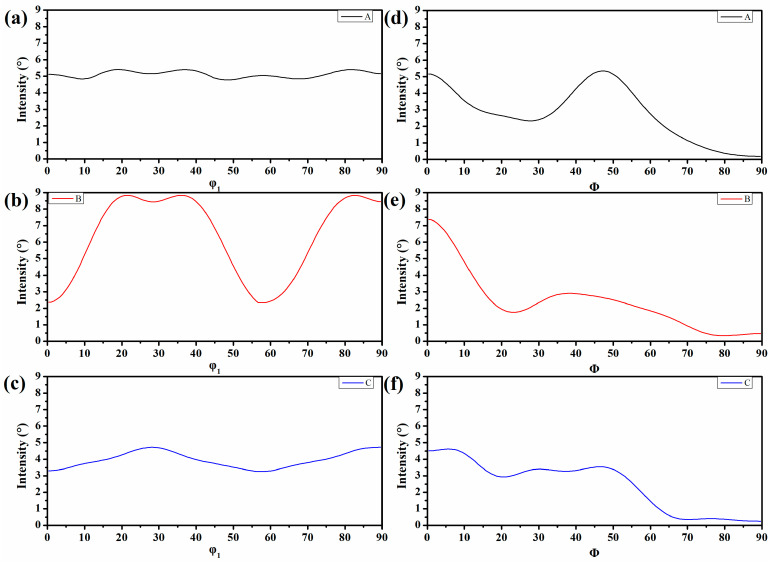
The exact intensity of the γ texture in specimen A (**a**), specimen B (**b**), specimen C (**c**), and the α texture (**d**–**f**) corresponding to (**a**–**c**), respectively.

It has been demonstrated that textures, which is the cause of anisotropy, supplied diversional directional material properties such as physical, mechanical, and chemical properties of the sample [34,35]. It is universally accepted that the γ texture is beneficial to formability, whereas the α texture attenuates it. Moreover, whether the texture was uniformly distributed would affect the deformation behavior as well. Generally, to enhance the deformation property, one should homogenize the distribution of the texture and fabricate intense γ texture. Thereby, ODF (orientation distribution function) images sectioned at $\varphi_{2}$ = 45° were employed (Figure 6). This suggests that evident textures were transformed in all these samples. To determine the exact types of the textures and their impacts on the HER, detailed intensities of the γ and α fiber texture are given in Figure 7.

It was shown that normal intense γ fiber and α fiber texture were transformed in all these samples. The rotated cube texture {100}[11] in α texture was also aggressively transformed in all these samples. The overall intensity of γ texture and α texture to specimens A and B was almost equivalent, as seen in Figure 7a,b, but they were weakened in specimen C. However, the distribution of the γ texture in specimen B was not homogeneous, wherein the intensity of the components composing the γ texture was dissimilar and it was uniformly distributed in specimen A (Figure 6a and Figure 7a). In addition, in specimen B, the α texture intensity was, on the whole, lower than that of specimens A and C, except for the rotated cube texture. Though the distribution of the γ texture in specimen A was more homogeneous than that in specimens B and C, it can be speculated that the HER of specimen B would be the best and should not be too different from specimen A as the γ fiber texture was beneficial to the formability and the α fiber texture was detrimental to it. However, the experimental results were inconsistent with them, implying the textures were not the controlling factors for the HER. 

### 3.5. Variant Pairing Analysis

**Figure 8:**
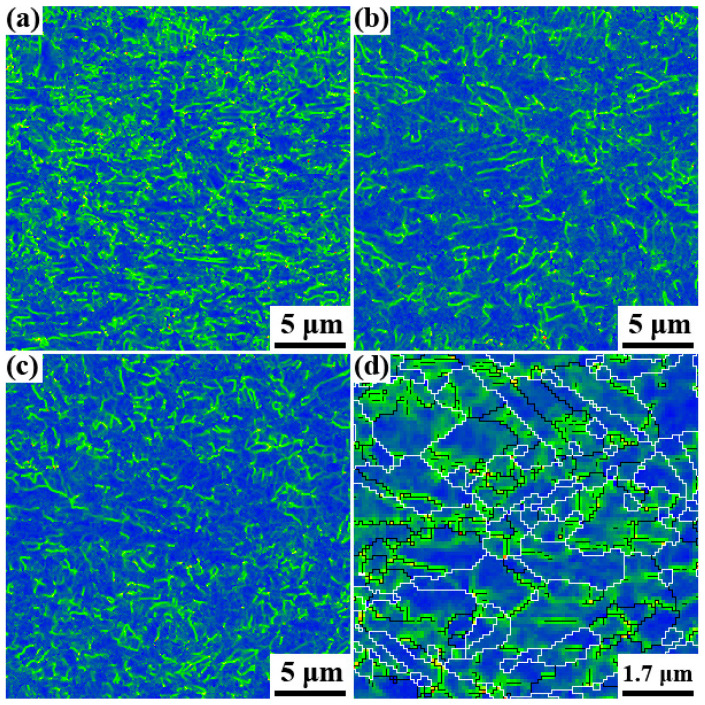
KAM map of specimen A (**a**), specimen B (**b**), specimen C (**c**), and a randomly chose enlarged KAM map superposed with boundaries of specimen A (**d**), wherein the black line depicted the boundaries between 2° to 15° and the white line represented the boundaries over 15°.

**Figure 9:**
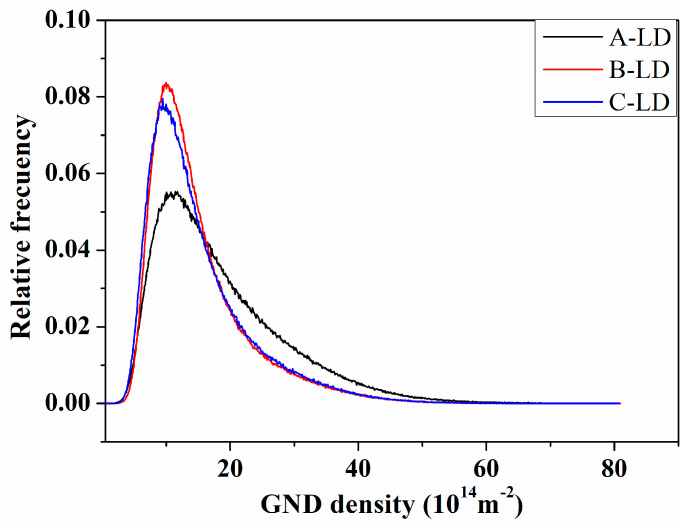
The statistics of the GND density.

**Figure 10:**
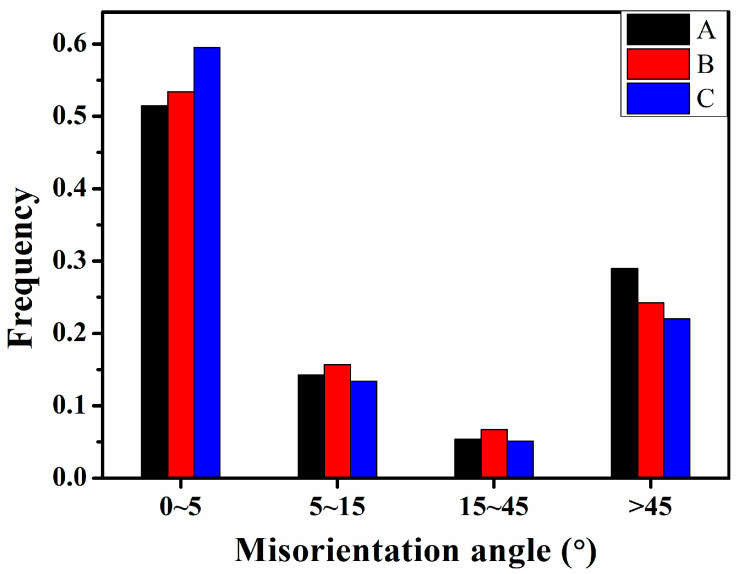
The relative density of grain boundaries.

As stated in Reference [36], the punching process accompanying the hole expansion test is primarily achieved by plastic deformation, which is governed by the collective motion of dislocations along the slip planes and slip directions. To increase the HER, the dislocation transmission ability across a grain boundary should be as strong as possible. Thus, the boundaries are discussed below. Wu et al. [24,25,26,27], Wang et al. [25], and Sun et al. [26,37] concluded that the mechanical properties such as the impact toughness were closely correlated to the dominating crystallography groups and the variant pair fractions. For this reason, it is reasonable to assume that the HER is related to the dominating crystallography groups and variant pairs fractions. Figure 8 shows the KAM (Kernel Average Misorientation) maps, from which it can be concluded that the geometrically necessary dislocation (GND) density in specimen A was exceedingly larger than that of specimens B and C. The statistic results in Figure 9 further confirmed this. Additionally, the geometrically necessary dislocations were primarily distributed along the LAGBs (low angle grain boundaries). Since the HER of specimen A was the best, it can be hypothesized that the ability to accommodate plastic deformation will be advanced if the relative density of HAGBs increased (Figure 10). The initial dislocations and the newly nucleated ones during stretch flanging operation could be transmitted through the GBs readily in specimen A. Thus, the GBs merits detailed emphasis.

It is well-known that the GBs originate from differential variant pairs for coherent or near-coherent phase transformation. To identify the GBs in terms of variant pairs exactly, the actual OR was firstly determined by the Miyamoto’s methodology [38], and Table 3 was the result. Table 4 tabulates the misorientation axes and angles of V1 to the other variants calculated from the K-S OR and the experimentally determined OR (actual OR), and the inter-variant boundary characteristics. 

**Figure 11:**
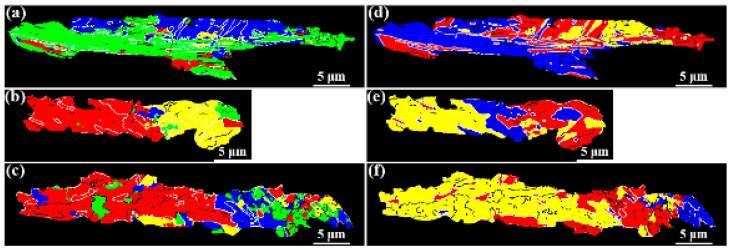
CP maps of specimen A (**a**), specimen B (**b**), specimen C (**c**), and the Bain maps (**d**–**f**) corresponding to (**a**–**c**), respectively. Black and white lines are low-angle (5° < θ < 15°) and high-angle (θ > 15°) boundaries.

As suggested in Table 4, the variants are classified into differential Bain groups and CP groups in crystallography. To clarify this, one individual prior austenite was randomly picked up in each specimen. The corresponded CP maps and Bain maps of these specimens are given in Figure 11, with black and white lines showing the low angle (5° < θ < 15°) and high angle (θ > 15°) grain boundaries, respectively. It was found that in specimens A and B, the CP group dominated the transformation, within which each CP region was composed of two or more Bain groups. The CP regions were massive in size with two or more Bain groups interactively distributed within them. Though the variants belonging to differential Bain groups formed some CP regions concurrently in specimen C, the variants belonging to differential CP groups had a higher chance of forming as a Bain region in specimen C than specimens A and B. Consequently, this led to a decrease in the density of high-angle grain boundaries. These results proved that the HER was closely related to the dominating crystallography groups. In the steels with high HER, the CP group dominated the transformation, and once the Bain grouping was dominated, the HER decreased. Moreover, in high-HER steels, the high-angle grain boundaries were mostly determined by the variants within the same CP group, and this phenomenon became the combination of those within the same CP group and those between differential CP groups in low-HER steel. However, in all these specimens, the variant pairs between differential CP groups all tended to form high0angle boundaries. The difference in the origin of high angle grain boundaries in the high HER steel and low HER steel was mostly attributed to the variant pair types within the same CP group and their fractions, which will be discussed next. 

**Figure 12:**
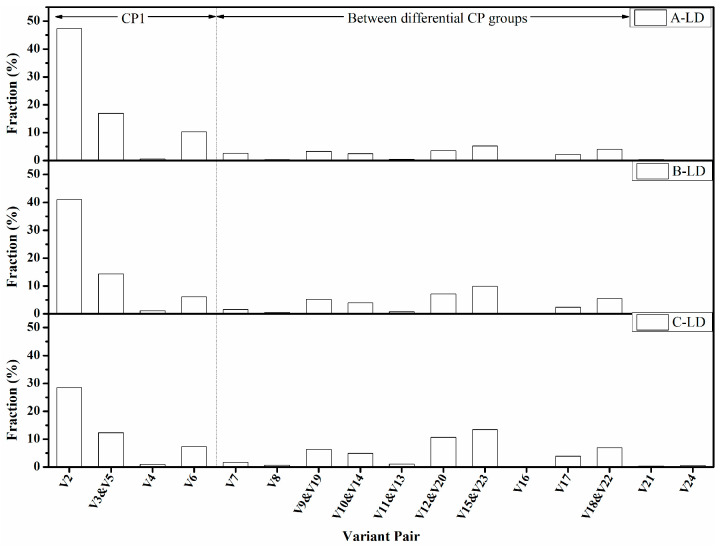
The fraction of the variant pairs constituting the angle boundaries in these specimens in the longitudinal direction.

**Figure 13:**
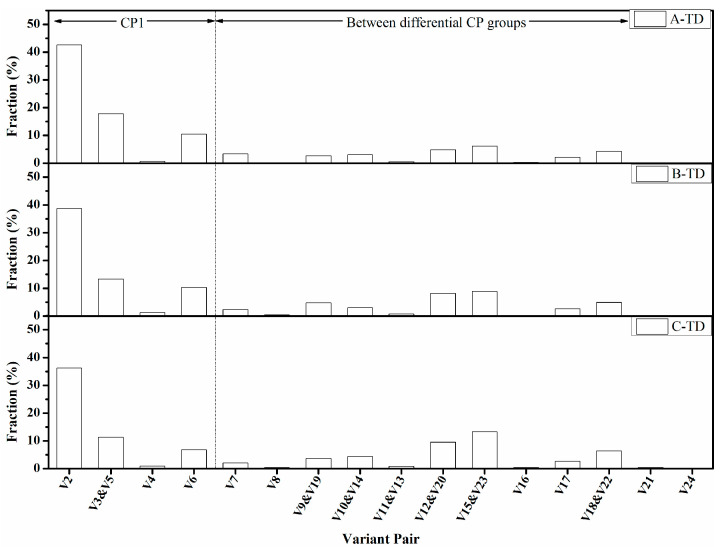
The fraction of the variant pairs constituting the angle boundaries in these specimens in the transverse direction.

Figure 12 showed the fraction of the variant pairs of these specimens in the longitudinal direction, with which a small tolerance angle of 1° to the above lattice rotations was employed to avoid overlapping. It was observed that the fractions of low-angle grain variant pairs (θ < 15°) of V1/V4, V1/V8, V1/V11, V1/V16, V1/V21, and V1/V24 were all low in all these specimens. However, there was a relationship between HER and the fraction of high-angle variant pairs. It was inferred that the high-angle variant pairs in specimens A and B were mostly within the same CP group. In contrast, specimen C had more variant pairs from differential CP groups. This result also confirmed that the domination of CP groups led to higher HER. The difference between these specimens was primarily in V1/V2, V1/V3, V1/V6, V1/V9, V1/V10, V1/12, V1/V15, V1/V17, and V1/V18 pairs, within which the V1/V2, V1/V15, and V1/V18 pairs had the maximum difference. The fraction of the V1/V2 pair in specimens A and B was extremely greater than that of specimen C, and for V1/V12 and V1/V15 pairs, their fractions were much lower. It should be noted that although the fraction trend of the V1/V3 pair was consistent with the HER, it was not the key factor responsible for HER because the dissimilarity between these specimens was significantly lower than for the V1/V2 pair. Interestingly, the V1/V6 pair within the same CP group was also special as the fraction of the V1/V6 pair in these specimens did not correspond to the HER, suggesting that it was not beneficial to the HER. The function of V1/V9, V1/V10, V1/V17, and V1/V18 pairs should be similar to V1/V12 and V1/V15 because their fractions exhibited a similar trend to V1/V12 and V1/V15 pair. Thus, it was proposed that the major factor concerning the HER of this steel was the fraction of V1/V2, V1/V12, and V1/V15 pair, wherein the V1/V2 pair was beneficial for stretch flangeability and V1/V12 and V1/V15 pairs were detrimental or at least not effective in enhancing HER. Similar results were discovered in the transverse direction (Figure 13), wherein the fraction of V1/V2 and V1/V3 pairs displayed a positive correspondence to the HER, and V1/V12, V1/V15, V1/V18 were more preferred as the HER decreased. It seems that the correlation between the HER and V1/V6 pair in the transverse direction was contradictory to that in the longitudinal direction. It would be confusing if just considering the fraction of the V1/V6 pair. Hence, we analyze the characteristics of the V1/V6 pair further in the next part of this article and validate that it was detrimental to HER.

As proposed in reference [36], the punching process accompanying the Hole Expansion Test was achieved by plastic deformation, which is governed by the collective motion of dislocations along the slip planes and slip directions. To increase the HER, the dislocation transmission ability across a grain boundary should be as high as possible. Ohmura [39] studied the effect of high-angle grain boundaries by employing a martensitic steel on dislocation motion and observed that the block boundary assisted the dislocation transmission across the high-angle grain boundaries. It also supported the above hypothesis as the block boundary originated from the high angle variant pairs within the same CP group (Table 4). Unfortunately, what exactly the block boundary belongs to in terms of variant pairs in Ohmura’s research was not identified because V1/V2, V1/V3, and V1/V6 were all block boundaries. They all had a chance to be the block boundary in Ohmura’s research. The function of V1/V2, V1/V3, and V1/V6 pairs for dislocation transmission could not be unambiguously determined in Ohmura’s observations. Hence, they merit further attention and are discussed next.

**Figure 14:**
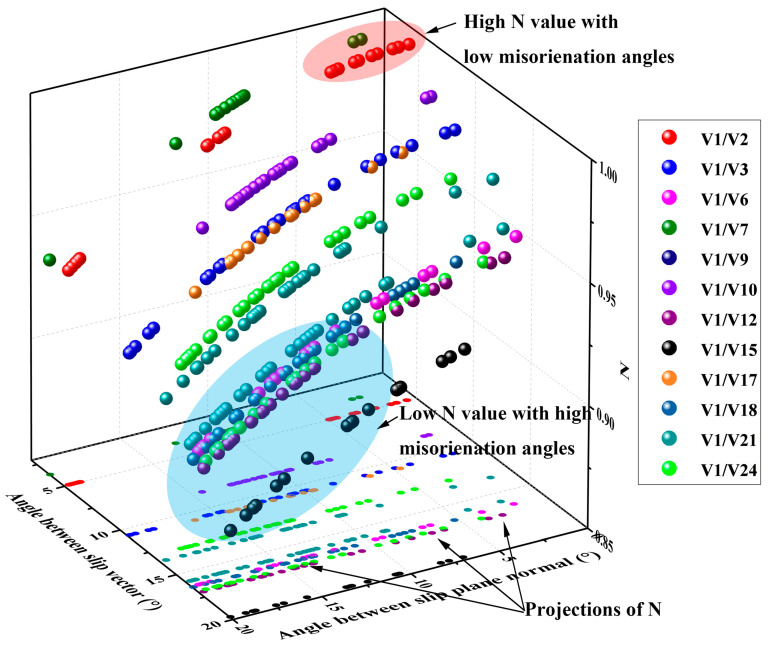
N values of slip systems in the angle range of 0° to 20° between slip plane normal and slip vector.

**Figure 15:**
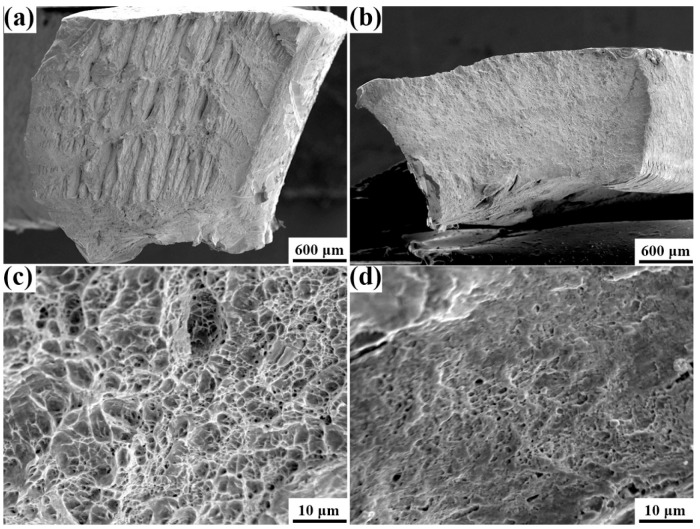
SEM fractographs of specimen A (**a**,**c**), specimen C (**b**,**d**).

As stated by Jordan [40], the common transmission factor, m’ = cos φ· cos k, which is a function of the angles between the slip vectors (φ) and slip plane normal (k), was effective in evaluating the dislocation slip transmission ability across a grain boundary. Higher m’ values indicated that grain boundaries were more penetrable. If the m’ equals 1, it means the grain boundary is transparent to dislocation slipping in this slip system. In body-centered cubic (BCC) metals such as α-Fe, a dislocation slip along the (111) directions was experimentally observed and verified by theoretical calculations [36]. For bcc metals, there are three possible slip families and {110}, {112}, and {123} planes along the [111] direction. However, previous studies [40,41,42,43,44] only considered the maximum N value from all the slip systems to evaluate the dislocation mobility. This is not appropriate because stretch flangeability is complex and several slip systems are most likely to be concurrently activated. Hence, we innovatively combined the maximum N value and the number of slip systems with high N values and low misorientation between slip plane normal and slip vectors and observed strong correspondence with the HER. Accordingly, the max m’ corresponding to the 16 variant pairs was calculated by the STABIX Matlab toolbox [45], which is listed in Table 5. The max m’ of the high angle V1/V2 pair and the low angle variant pairs of V1/V4, V1/V7, and V1/V8 were 1.00, implying that these boundaries were completely transparent, while the other high angle-variant pairs, namely V1/V9, V1/V12, V1/V15, and V1/V18, were partially transmittable. This result was consistent with the HER. In particular, the max m’ factors of the V1/V2 pair to the three slip families were all 1.0, demonstrating that the V1/V2 pair was especially favorable to the dislocation motion. Extraordinary promotion of slip transfer for V1/V2 pair was expected and as a result improved the HER. Hence, increasing the fractions of V1/V2, V1/V4, V1/V7, and V1/V8 pair, particularly the V1/V2 pair, would increase the HER. To enhance the stretch flangeability, the other high angle variant pairs and low angle variant pairs should be prevented from being transformed. It should be noted that the m’ factor for the V1/V6 pair in all the three slip families was low, proving that the resistance to dislocation slip transmission was large, which was detrimental to HER. On the contrary, the V1/V7 pair from the differential CP group was beneficial for dislocation transmissions. The N values (Figure 14) in the angle range of 0° to 20° to the slip plane normal and slip vector for all these forty-eight slip systems showed that V1/V2 pair exhibited the largest magnitude of high-N-value slip system. Furthermore, the majority of these slip families with high N values in the V1/V2 pair had lower misorientation angles between slip plane normal and slip vector, which was [0°, 5°]. These slip systems can be perfectly aligned for the activation of slip during stretch flanging. The N values of variant pairs V1/V6, V1/V12, V1/V15, and V1/V18 were all intriguingly lower than V1/V2, which also confirmed the aforementioned hypothesis. Moreover, the misorientation angles between the slip plane normal and slip directions to these variant pairs were all large. The majority of them exceeded 10° in the slip plane normal and 16° in the slip direction, indicating the slip systems were not well aligned and could not effectively promote dislocation transmission. Although the authors could not determine for sure, it could be supposed that the V1/V7 and V1/V10 pair might also be favorable in improving HER as they reported an adverse trend in the fraction of the HER, and their N values were large and the misorientation angles were low. Therefore, making the V1/V2 and V1/V3 pairs more pronounced in both longitudinal and transverse directions guaranteed that the dislocation can be transmitted in both directions, thereby improving the HER. The preference for the V1/V12 and V1/V15 pairs in specimen C for both longitudinal and transverse directions exacerbated the HER. This would exert a vital effect on the morphology of the fracture, as reference [46,47] have suggested; for the GBs that were impenetrable to dislocation motions, they would cause cleavage fracture as the dislocation slippery was prevented and these dislocations could not slip long. When they favor dislocation transmissions, it would change into dimple fractures as these dislocations could move a long journey and as a result enhance the plasticity and HER. Thus, the fracture to specimen A with more V1/V2 pairs should primarily be a dimple, while it would be more of a cleavage for specimen C as it possessed more V1/V12 and V1/V15 pairs, as confirmed in Figure 15. Hence, to improve the stretch flangeability performance, one should increase the fraction of the V1/V2 and V1/V3 pairs, especially the V1/V2 pair, whereas the V1/V6, V1/V9, V1/V12, V1/V15, and V1/V18 pair should be prevented from being transformed.

## 4. Conclusions

The crystallographic characteristics played a critical role in evaluating the mechanical properties as reported in previous works. A thorough comprehension of the HER consisted the volume fraction of retained austenite, the M/A (martensite austenite constituents) fraction, the precipitations, the grain diameter of the ambient microstructure, the density of high angle grain boundaries, the texture, and especially the variant pairing characteristics. It was concluded, in this steel that:

(1) The contributions of the volume fraction of retained austenite, the M/A (martensite austenite constituents) fraction, the precipitations, the grain diameter of the ambient microstructure, the density of high angle grain boundaries, and the texture to the HER were limited. They were not the controlling factors for the HER.
(2) The HER was closely correlated to the dominating crystallography groups. Whether or not the high-angle-variant pairs were transformed from the same CP group played a critical role in determining the HER, which was ascribed to that the variants within the same CP group sharing a common habit plane. For the high-HER steel, the CP groups dominated the transformation, while it was the CP groups and Bain groups together that determined the transformation for low-HER steel.
(3) The types of variant pairs and their fractions contributed significantly to the HER. High-angle variant pairs of V1/V2, V1/V3, V1/V7, and especially the V1/V2 pair were beneficial to the HER, whereas the impacts of high-angle-variant pairs of V1/V6, V1/V9, V1/V10, V1/V12, V1/V15, V1/V17, and V1/V18 were limited or even detrimental. This result showed that the variant pair type and their fractions might account for most the variation of HER in this steel. The variant pairs deserve further attention, and they provide new insight into the actions in enhancing the HER.
(4) The effects of the variant pairs to HER were attributed to the differential dislocation transmission ability across GBs. The V1/V2 pair was specialized in supplying a slip passage for dislocation transmission across a grain boundary with little resistance, whereas the dislocation transmission ability across the inter-variant boundary of V1/V9, V1/V12, and V1/V15 was particularly poor.

## Figures and Tables

**Table 1:** Chemical compositions of the three steel plates (wt.%).

C	Si	Mn	P	S	Cr	Ti
0.06	0.56	1.71	0.012	0.002	0.32	0.1

**Table 2:** Tensile properties of the three steel plates.

Sample	Yield Strength (MPa)	Tensile Strength (MPa)	Total Elongation (%)
A	716	871	14.5
B	804	908	12.5
C	812	910	9.5

**Table 3:** Orientation relationships (OR) of these specimens.

		Euler Angle (φ_1_, Φ, φ_2_)
Exact K-S OR		114.2°, 10.5°, 204.2°
Actual OR	A	120.8°, 9.2°, 195.8°
	B	119.6°, 9.1°, 196.9°
	C	121.4°, 9.3°, 195.1°

**Table 4:** Misorientation axes and angles of V1 to the other variants calculated from the experimentally determined OR (actual OR) and the inter-variant boundary characteristics.

Variant	Parallel Plane	Parallel Direction	Rotation Angle/axis to V1				CP Group	Bain Group	Boundary Type
			Exact K-S OR	Specimen A	Specimen B	Specimen C			
V1	(111)_γ_//(011)_α_	[−101]_γ_//[−1−11]_α_	-	-	-	-	CP1	B1	Block
V2		[−101]_γ_//[−11−1]_α_	60.0°/[11−1]	60.2°/[0.53, 0.55, −0.64]	60.2°/[−0.55, −0.53, 0.64]	60.2°/[0.53, 0.64, −0.55]		B2	Block
V3		[01−1]_γ_//[−1−11]_α_	60.0°/[011]	60.0°/[−0.03, 0.70, −0.71]	60.0°/[−0.51, 0.44, −0.72]	60.0°/[−0.02, 0.70, −0.71]		B3	block
V4		[01−1]_γ_//[−11−1]_α_	10.5°/[0−1−1]	5.7°/[0.00, −0.61, −0.79]	5.8°/[0.00, −0.83, −0.56]	5.7°/[0.00, −0.63, −0.77]		B1	Sub-Block
V5		[1−10]_γ_//[−1−11]_α_	60.0°/[0−1−1]	60.0°/[0.70, −0.71, 0.03]	60.0°/[0.47, 0.72, −0.51]	60.0°/[0.70, 0.71, −0.02]		B2	Block
V6		[1−10]_γ_//[−11−1]_α_	49.5°/[011]	54.4°/[0.04, 0.71, 0.71]	54.3°/[−0.04, 0.71, 0.71]	54.4°/[0.03, 0.71, 0.71]		B3	Block
V7	(1–11)_γ_//(011)_α_	[10−1]_γ_//[−1−11]_α_	49.5°/[−1−11]	51.2°/[−0.52, −0.61, 0.61]	51.6°/[−0.61, −0.52, 0.61]	50.8°/[−0.61, −0.52, 0.61]	CP2	B2	Packet
V8		[10−1]_γ_//[−11−1]_α_	10.5°/[11−1]	9.7°/[0.68, 0.68, −0.25]	9.3°/[0.69, 0.69, −0.24]	10.0°/[0.68, 0.68, −0.26]		B1	Packet
V9		[−1−10]_γ_//[−1−11]_α_	50.5°/[−103−13]	52.4°/[−0.65, 0.21, −0.73]	52.4°/[−0.66, 0.21, −0.72]	52.3°/[−0.65, 0.21, −0.73]		B3	Packet
V10		[−1−10]_γ_//[−11−1]_α_	50.5°/[−7−55]	51.2°/[−0.69, −0.45, 0.57]	51.3°/[−0.69, −0.45, 0.57]	51.0°/[−0.69, −0.45, 0.56]		B2	Packet
V11		[011]_γ_//[−1−11]_α_	14.9°/[1351]	13.0°/[0.87, 0.49, 0.06]	12.9°/[0.88, 0.47, 0.07]	13.2°/[0.87, 0.50, 0.06]		B1	Packet
V12		[011]_γ_//[−11−1]_α_	57.2°/[−356]	58.0°/[−0.66, 0.19, −0.73]	58.0°/[−0.66, 0.18, −0.73]	57.9°/[−0.66, 0.19, −0.73]		B3	Packet
V13	(–111)_γ_//(011)_α_	[0−11]_γ_//[−1−11]_α_	14.9°/[5−13−1]	13.0°/[0.49, −0.87, −0.06]	12.9°/[0.47, −0.88, −0.06]	13.2°/[0.50, −0.87, −0.06]	CP3	B1	Packet
V14		[0−11]_γ_//[−11−1]_α_	50.5°/[−55−7]	51.2°/[−0.57, 0.45, −0.69]	51.3°/[−0.57, 0.45, −0.69]	51.0°/[−0.56, 0.45, −0.69]		B3	Packet
V15		[−10−1]_γ_//[−1−11]_α_	57.2°/[−6−25]	56.6°/[−0.71, −0.24, 0.66]	57.0°/[−0.71, −0.66, 0.25]	56.3°/[−0.71, −0.24, 0.66]		B2	Packet
V16		[−10−1]_γ_//[−11−1]_α_	20.6°/[11−11−6]	16.2°/[0.69, −0.69, −0.23]	16.3°/[0.69, −0.69, −0.21]	16.4°/[0.69, −0.69, −0.24]		B1	Packet
V17		[110]_γ_//[−1−11]_α_	51.7°/[−116−11]	51.4°/[−0.65, 0.39, −0.65]	52.3°/[−0.66, 0.35, −0.66]	51.3°/[−0.65, 0.39, −0.65]		B3	Packet
V18		[110]_γ_//[−11−1]_α_	47.1°/[−24−102]	51.2°/[−0.70, −0.28, 0.65]	51.3°/[−0.71, −0.28, 0.65]	50.9°/[−0.70, −0.27, 0.66]		B2	Packet
V19	(11–1)_γ_//(011)_α_	[−110]_γ_//[−1−11]_α_	50.5°/[−31310]	52.4°/[−0.21, 0.73, 0.65]	52.4°/[−0.21, 0.72, 0.66]	52.3°/[−0.21, 0.73, 0.65]	CP4	B3	Packet
V20		[−110]_γ_//[−11−1]_α_	57.2°/[36−5]	58.0°/[−0.19, −0.73, −0.66]	58.0°/[0.19, 0.73, −0.66]	58.0°/[−0.19, −0.73, −0.66]		B2	Packet
V21		[0−1−1]_γ_//[−1−11]_α_	20.6°/[30−1]	18.0°/[0.99, 0.00, −0.15]	17.8°/[0.99, 0.00, −0.14]	18.4°/[0.99, 0.00, −0.16]		B1	Packet
V22		[0−1−1]_γ_//[−11−1]_α_	47.1°/[−102124]	51.2°/[−0.28, 0.65, 0.70]	51.3°/[−0.28, 0.65, 0.71]	50.9°/[−0.27, 0.66, 0.70]		B3	Packet
V23		[101]_γ_//[−1−11]_α_	57.2°/[−2−5−6]	56.6°/[−0.24, −0.66, −0.71]	56.8°/[−0.25, −0.71, −0.66]	56.3°/[−0.24, −0.66, −0.71]		B2	Packet
V24		[101]_γ_//[−11−1]_α_	21.1°/[9−40]	18.4°/[0.96, −0.27, 0.00]	18.3°/[0.96, −0.29, 0.00]	18.7°/[0.96, −0.26, 0.00]		B1	Packet

**Table 5:** The max m’ corresponding to the 16 variant pairs calculated by the STABIX Matlab toolbox.

Variant Pairing to V1	Misorientation [°]	Max m’		
		{110}[111]	{112}[111]	{123}[111]
V2	60.2	1.00	1.00	1.00
V3	60.0	0.98	0.98	0.96
V4	5.7	0.99	0.99	1.00
V6	54.4	0.96	0.95	0.96
V7	51.2	0.99	0.99	0.99
V8	9.7	0.99	0.99	0.99
V9	52.4	0.92	0.93	0.92
V10	51.2	0.97	0.98	0.98
V11	13.0	0.97	0.97	0.97
V12	58.0	0.95	0.95	0.96
V15	56.6	0.93	0.94	0.93
V16	16.2	0.96	0.96	0.99
V17	51.4	0.97	0.96	0.97
V18	51.2	0.94	0.93	0.94
V21	18.0	0.95	0.95	0.96
V24	18.4	0.95	0.95	0.96

## Data Availability

No new data were created or analyzed in this study. Data sharing is not applicable to this article.
